# Pig Farmers’ Homes Harbor More Diverse Airborne Bacterial Communities Than Pig Stables or Suburban Homes

**DOI:** 10.3389/fmicb.2018.00870

**Published:** 2018-05-01

**Authors:** Ditte V. Vestergaard, Gitte J. Holst, Ioannis Basinas, Grethe Elholm, Vivi Schlünssen, Allan Linneberg, Tina Šantl-Temkiv, Kai Finster, Torben Sigsgaard, Ian P. G. Marshall

**Affiliations:** ^1^Section for Environment, Occupation and Health, Department of Public Health, Aarhus University, Aarhus, Denmark; ^2^Section for Microbiology, Department of Bioscience, Aarhus University, Aarhus, Denmark; ^3^Centre for Human Exposure Science, Institute of Occupational Medicine, Edinburgh, United Kingdom; ^4^National Research Centre for the Working Environment, Copenhagen, Denmark; ^5^Department of Clinical Experimental Research, Rigshospitalet, Denmark; ^6^Department of Clinical Medicine, Faculty of Health and Medical Sciences, University of Copenhagen, Copenhagen, Denmark; ^7^Research Centre for Prevention and Health, Rigshospitalet Glostrup, Glostrup, Denmark

**Keywords:** bacteria, pig stables, built environment, microbiome, airborne bacteria, 16S rRNA gene

## Abstract

Airborne bacterial communities are subject to conditions ill-suited to microbial activity and growth. In spite of this, air is an important transfer medium for bacteria, with the bacteria in indoor air having potentially major consequences for the health of a building’s occupants. A major example is the decreased diversity and altered composition of indoor airborne microbial communities as a proposed explanation for the increasing prevalence of asthma and allergies worldwide. Previous research has shown that living on a farm confers protection against development of asthma and allergies, with airborne bacteria suggested as playing a role in this protective effect. However, the composition of this beneficial microbial community has still not been identified. We sampled settled airborne dust using a passive dust sampler from Danish pig stables, associated farmers’ homes, and from suburban homes (267 samples in total) and carried out quantitative PCR measurements of bacterial abundance and MiSeq sequencing of the V3–V4 region of bacterial 16S rRNA genes found in these samples. Airborne bacteria had a greater diversity and were significantly more abundant in pig stables and farmers’ homes than suburban homes (Wilcoxon rank sum test *P* < 0.05). Moreover, bacterial taxa previously suggested to contribute to a protective effect had significantly higher relative and absolute abundance in pig stables and farmers’ homes than in suburban homes (ALDEx2 with *P* < 0.05), including Firmicutes, Peptostreptococcaceae, Prevotellaceae, Lachnospiraceae, Ruminococcaceae, *Ruminiclostridium*, and *Lactobacillus*. Pig stables had significantly lower airborne bacterial diversity than farmers’ homes, and there was no discernable direct transfer of airborne bacteria from stable to home. This study identifies differences in indoor airborne bacterial communities that may be an important component of this putative protective effect, while showing that pig stables themselves do not appear to directly contribute to the airborne bacterial communities in the homes of farmers. These findings improve our understanding of the role of airborne bacteria in the increasing prevalence of asthma and allergy.

## Introduction

Bacteria in indoor air typically stem both from buildings’ occupants and outside air (Hospodsky et al., 2012; Qian et al., 2012; Meadow et al., 2014; Adams et al., 2015a). Restricted water and nutrient availability make the aerial environment poorly conducive to microbial activity and growth (Després et al., 2012). In spite of these inhibiting factors, air acts as a transfer medium between various sources and sinks of bacteria including humans, animals, plants, soil, and water (Šantl-Temkiv et al., 2018). Exposure to airborne bacteria has consequences for the health of building occupants. For example, the airborne microbial community has been shown to differentiate environments promoting and inhibiting the development of asthma and allergy (Haahtela et al., 2015). Ege et al. (2011) showed that increased diversity of microorganisms is associated with lower asthma and atopy prevalence in children living on farms. Lower allergy risk has been related to differences in airborne bacterial composition, such as higher numbers of Gram-positive bacteria (Pakarinen et al., 2008). Lynch et al. (2014) found that healthy children were exposed to dust containing a higher fraction of specific bacterial taxa, such as families Prevotellaceae, Lachnospiraceae, and Ruminococcaceae, in their home environments than in the house dust of homes with of children with atopy or wheeze. Clostridium cluster XI (also known as Peptostreptococcaceae; Galperin et al., 2016) has also been associated with protection against asthma (Pekkanen et al., 2018). These studies suggest that exposure to a specific set of airborne microbial taxa may help to reduce the risk of development of asthma and allergies.

The prevalence of asthma and allergies is a widespread and growing problem in affluent areas of the world (Latvala et al., 2005; Eder et al., 2006). Being born and/or raised on a farm has been shown to protect against atopic asthma and allergies (Braun-Fahrländer et al., 1999, 2002; Riedler et al., 2001; Portengen et al., 2002; Radon et al., 2006; Illi et al., 2012; Elholm et al., 2013, 2016). Pig farmers have also been reported to have less atopic sensitization than the general population (Portengen et al., 2005). On the other hand, working in pig stables has been linked to detrimental health impacts, including non-atopic asthma (Eduard et al., 2004) and chronic obstructive pulmonary disease (COPD; Monsó et al., 2004; Eduard et al., 2009), both of which may be a result of airborne exposures to pathogen-associated molecular patterns (PAMPS; Sigsgaard et al., 2005). Moreover, potentially infectious airborne *Staphylococcus aureus* is known to be present in pig stable air (Masclaux et al., 2013). There is a need to distinguish between the beneficial and the detrimental factors of working and living on farms with particular impact on farm workers’ health.

The exact mechanisms by which airborne bacteria may confer protection against allergy and asthma are unknown, but there is growing evidence supporting a link between exposure to certain groups of microbes and the development of allergies and asthma. A specific mouse gut microbiota signature has been linked to the development of allergic sensitization and anaphylaxis (Noval Rivas et al., 2013). In humans, the gut microbiota in allergic individuals was characterized by a smaller fraction of the bacterial order Clostridiales (Hua et al., 2016). Furthermore, airway bacterial communities in asthma patients are distinct from non-asthmatic individuals (Hilty et al., 2010; Huang et al., 2011). The positive effect of specific bacterial types has been related to the stimulation of regulatory T cells (Tregs) by beneficial bacteria. These Tregs inhibit allergen-specific T cells by secreting inhibitory cytokines (Rook and Brunet, 2005). Certain species of microbes, such as the gut commensal bacterium *Bacteroides fragilis*, have been shown to stimulate Tregs (Smits et al., 2005; Round and Mazmanian, 2010). Early-life oral exposure to certain Gram-positive bacterial species, such as *Clostridium* (a.k.a. *Ruminiclostridium*) *leptum* (Li et al., 2012, 2014) and *Lactobacillus reuteri* (Forsythe et al., 2007; Karimi et al., 2009), has been shown to induce protection against asthma in mice through the induction of Tregs. In most cases, the property that separates these “protective” strains from other strains has not been identified, except in the case of *B. fragilis* where the effect is caused by capsular polysaccharide A (Round and Mazmanian, 2010). The strains used in these studies were chosen based on their widespread role as commensal gut bacteria, but microbial community analysis studies have shown that there may be a much wider range of bacteria that confer this positive effect. It is possible that the protection against asthma and allergy in farming environments is a result of exposure to airborne bacteria.

Our study is an extension of the SUS (“Sund Stald” – healthy stable) project that was initiated in 1992 with the purpose of investigating respiratory health hazards among farmers. The study used students from Danish farming schools as the test population of interest, with Danish military conscripts as a control group (Sigsgaard et al., 1997). The study found a significantly reduced prevalence of atopy among young Danish farmers, especially among those raised on farms (Portengen et al., 2002). SUS12 was the follow-up study conducted in 2007/2008, where the participants of the original SUS study were reexamined (Elholm et al., 2010). As part of SUS12 study, settled dust samples were collected in pig stables and in farmers’ bedrooms over a 14-day period using an electrostatic dust collector (EDC). The same sampling method was used in a separate study collecting dust in bedrooms in a suburban area in the Western part of Copenhagen (Holst, 2016). We used these suburban bedroom samples as a point of comparison for the farm-based samples. To obtain deeper qualitative and quantitative insights in the airborne microbial communities at the three types of sampling locations, we chose to use cultivation-independent methods, such as quantitative PCR and MiSeq 16S rRNA gene amplicon sequencing. Previously, total bacterial concentrations in pig stables have been shown to be 100–1000 times higher than the cultivable bacterial fractions (Nehme et al., 2008).

There have been a number of other cultivation-independent studies addressing the airborne microbial community of pig stables (Nehme et al., 2008, 2009; Hong et al., 2012; Kristiansen et al., 2012; Boissy et al., 2014; Kumari and Choi, 2014, 2015), but this is the largest study so far in terms of the number of pig stables studied (43 facilities). It is also unique to have samples from farmers’ homes and suburban homes collected and analyzed with identical methods. Comparing airborne bacteria in pig stables, farmers’ homes, and suburban homes may help identify the differences in bacterial abundance, diversity, and community composition. It might also provide insight into how differences in environmental bacteria may be responsible for the different degrees of asthma and allergy development that develop in people exposed to different environments.

## Materials and Methods

### Dust Sampling

Dust was collected from the air using a passive airborne dust collection method referred to as an electrostatic dust fall collector (EDC). The EDC has been evaluated and tested in a similar environment and has proven to be a valid method for dust collection (Noss et al., 2008; Kilburg-Basnyat et al., 2016a,b). The EDC has an electrostatic cloth attached to it with an exposure area of 0.0209 m^2^ for dust to settle. Sampling took place in farmers’ homes and pig stables in Jutland, Denmark, in winter (43 samples from pig stables and 43 samples from farmers’ homes collected from November to April) and summer (40 samples from pig stables and 41 samples from farmers’ homes collected from May to October), with season cutoffs chosen to reflect major average monthly temperature shifts. This sampling was part of a previous study (Basinas et al., 2012; Schlünssen et al., 2015). The decision to focus on farms in Jutland was driven by the fact that the majority of Danish livestock production (around 80%) takes place in Jutland (Statistics Denmark, 2018). For comparison with suburban homes, sampling was carried out in Greater Copenhagen in winter (50 samples collected from November to February) and summer (50 samples collected from June to August). These samples were also obtained from a previous study (Holst, 2016). Each EDC was placed approximately 1.5 m above ground to reproduce typical breathing exposure conditions. The desired sampling collection time was 14 days, with the actual sampling time varying (Supplementary Figure 1). In the suburban and farm homes, the EDCs were placed in the bedroom and in the pig stable, the EDCs were placed in the area where the farmers spent most of their time. EDCs were stored at -80°C until being used for DNA extraction in this study.

### Dust Extraction From Filters

The EDC filters were handled as described previously (Adams et al., 2015b). They were carefully placed into a sterile stomacher bag and mixed with 20 ml extraction buffer consisting of 20 ml pyrogen-free water (PURELAB Ultra, ELGA, High Wycombe, United Kingdom) with 0.05% Tween-20. The sample was processed in a stomacher (Star Blender LB 400, Seward, Worthing, United Kingdom) for 10 min at maximum speed. The fluid was collected in a 50 ml falcon tube and kept on ice for <1 h. This procedure was repeated until a total volume of 40 ml was extracted from the filter. Following dust extraction, the samples were centrifuged at 4700 × *g* for 15 min at 5°C. The supernatant was removed and the pellet was suspended in the tween extraction buffer to a total volume of 1.5 ml. Negative control extractions were carried out using clean filters (2× extractions). The extracted dust samples were stored at -20°C until DNA extraction.

### DNA Extraction

The PowerLyzer PowerSoil DNA Isolation kit (now known as the DNeasy PowerLyzer PowerSoil Kit, MO BIO Laboratories, a Qiagen Company) was used to extract DNA from the extracted dust pellets. The kit was used according to manufacturers’ instructions with the following refinements: the bead-beating step was carried out in a TissueLyser bead-beating machine for 2 × 5 min at 50 s^-1^. The relatively long bead-beating step was chosen due to an expectation that much of the pig stable airborne bacterial community would consist of spores (Kristiansen et al., 2012). Immediately following bead beating, the samples were centrifuged at 13,000 × *g* for 5 min at room temperature. Bead beating and centrifugation were carried out twice for each sample for a total of 4 × 5 min bead beating. Additional negative control extractions were carried out using dust extraction buffer (2× extractions) and pyrogen-free water from the same source as used for the buffer (2× extractions).

### Quantitative PCR

Ten samples from each location type/season combination (three location types – farmers’ home, suburban home, and pig stable, two seasons – winter and summer, 60 samples total) were randomly selected for quantitative PCR to quantify bacterial abundance, and additional samples from each location type/season combination were selected from those with sampling times less than or greater than the target 14 days. qPCR primers were Bac908F (5′-AAC TCA AAK GAA TTG ACG GG-3′) and Bac1075R (5′-CAC GAG CTG ACG ACA RCC-3′) (Ohkuma and Kudo, 1998). The PCR reaction mixture contained 10 μl SYBR Green 1 Master-2x, 2 μl bovine serum albumin (BSA; 10 mg/ml), 1 μl forward primer, 1 μl reverse primer (10 pmol/μl), 4 μl dH_2_O, and 2 μl template DNA. The thermal cycling conditions were one cycle of initial denaturation for 5 min at 95°C, followed by 45 cycles at 95°C for 30 s, 56°C for 30 s, 72°C for 20 s, and 80°C for 7 s. A plasmid containing a full-length 16S rRNA gene related to *Sphingomonadales* was used to create a standard curve that was prepared fresh for every qPCR thermal cycling run. Thermal cycling and fluorescence measurements were carried out using an MX3005p qPCR machine (Agilent, Santa Clara, CA, United States). Homogeneity of amplicon sizes was confirmed following each qPCR run using melt curves, with all qPCR runs showing homogeneous amplicon sizes as expected. Significance of the differences in abundance of bacteria in different location types and seasons was determined using the Wilcoxon Rank Sum test implemented in the “wilcox.test” function in R version 3.3.0.

### MiSeq 16S rRNA Gene Amplicon Sequencing

The 16S rRNA gene was amplified from 296 samples (267 samples, 10 negative control samples, and 19 technical replicates where the same DNA extract was used for multiple PCR reactions) with bacteria-specific primers [Bac341F (5′-CCT ACG GGN GGC WGC AG-3′) and Bac805R (5′-GAC TAC HVG GGT ATC TAA TCC-3′)] targeting the V3 and V4 regions (Klindworth et al., 2013). Six negative control samples were from dust extraction and sample-free DNA extraction and four negative control samples were template-free PCR controls, with one template-free negative control added to each PCR batch. The 16S rRNA gene amplification steps were prepared as described by the Illumina protocol (16S Metagenomic Sequencing Library Preparation, Part # 15044223 Rev. B) with several modifications.

The PCR mixture contained 2–5 μl template DNA, 2 × KAPA HiFi Hotstart polymerase (KAPA Biosystems, Wilmington, MA, United States), 0.2 μM forward primer, 0.2 μM reverse primer, and BSA (4 g/L); 2 μL template DNA was used for pig stable samples, 3 μL template DNA was used for farmers’ home samples, and 5 μL template DNA was used for suburban home samples. These template amounts were chosen to maximize template abundance while still allowing the reaction to proceed without inhibition. This variation in template volume probably reflects different concentrations of bacteria and PCR inhibitors in the different environments. The thermal cycling was performed in the following steps: an initial denaturation at 95°C for 3 min, 25 cycles with denaturation at 95°C for 30 s, annealing at 55°C for 30 s, elongation at 72°C for 30 s, and a final elongation at 72°C for 5 min. The PCR products were cleaned using 30 μl AMPure XP magnetic beads. The second PCR incorporated the Illumina overhang adaptors. The PCR conditions were the same as for the first PCR, albeit without added BSA and with only 10 amplification cycles instead of 25. The product was again cleaned, this time with 20 μl AMPure XP beads as described in the protocol. The third PCR included Nextera XT Index primers from the Nextera XT Index kit. Each reaction contained 2.5 μl Index primer 1 (N7XX) and 2.5 μl Index primer 2 (S5XX), 12.5 μl KAPA HiFi HotStart ReadyMix, and 5 μl dH_2_O. The PCR thermal cycling program was the same as described above; however, this time only eight amplification cycles were used. The PCR product was cleaned with 56 μl AMPure XP beads as described in the Illumina 16S protocol.

The concentrations of PCR product were measured using a Quant-iT^TM^ dsDNA BR assay kit on a FLUOstar Omega fluorometric microplate reader (BMG LABTECH, Ortenberg, Germany) and diluted to a concentration of approximately 3 ng/μl DNA and then pooled together. The pools were measured using the Quant-iT^TM^ dsDNA BR assay kit on a Qubit fluorometer (Thermo Fisher Scientific, Waltham, MA, United States) before sequencing using a MiSeq sequencer (Illumina, San Diego, CA, United States).

### Bioinformatics

Sequence data analyses were performed using mothur version 1.39.3 (Schloss et al., 2009). Sequences belonging to the forward and reverse read libraries were merged together and only sequences with a length of 400–500 base pairs, which was the expected amplicon length based on the primers used, were accepted for further analysis. The sequences were aligned to a SILVA database version 128 (Quast et al., 2013). Sequences that failed to align across the expected 16S rRNA gene region were removed, and remaining sequences were trimmed to the expected region. Sequence error was reduced using mothur’s “pre.cluster” command, with up to 4 bp differences permitted per pre-cluster. Chimeric sequences were detected and removed using the UCHIME algorithm (Edgar et al., 2011) from within mothur. The sequences were taxonomically classified using the default Wang method to the SILVA 128 database and clustered into operational taxonomic units (OTU) using VSEARCH abundance-based greedy clustering (AGC; Rognes et al., 2016) with a similarity threshold of 97%. Singleton OTUs (OTUs occurring only once across all samples) were removed from the dataset (0.25–16.13% reads removed, median 0.47%). A consensus taxonomy for each OTU was found using the “classify.otu” function within mothur with default settings. OTUs classified as domain “Archaea,” “Eukaryota,” “unknown,” class “Chloroplast,” and family “Mitochondrion” were removed from the dataset (0.00–8.72% reads removed, median 0.47%).

Putative contaminant OTUs were removed from the dataset based on the following criterion: if, for a given OTU, the maximum number of reads out of all negative control samples was greater than the maximum number of reads out of all samples, then this was considered a true contaminant OTU and removed from the data set (0.15–72.23% reads removed, median 5.24%). This was done to avoid misidentification of misassigned sample reads (assigned to negative control reads due to tag-switching as described in Wright and Vetsigian, 2016) as a true part of the negative control.

Diversity estimates (richness and Shannon index) were determined using the “estimate_richness” function in phyloseq version 1.16.2 (McMurdie and Holmes, 2013) with R version 3.3.0. Significance of the difference in richness between different seasons and location pairs using the Wilcoxon rank sum test implemented in the “wilcox.test” function in R. Significance of differential abundance for phyla, orders, and families was determined using the ALDEx2 package version 1.4.0 (Fernandes et al., 2013, 2014).

NMDS ordination was carried out in R version 3.3.0 using phyloseq version 1.16.2. Technical replicates and samples with fewer than 8000 reads were removed from the analysis and 8000 reads were randomly subsampled from each of the remaining samples. Ordination was carried out using the “ordinate” function in phyloseq, using a Bray–Curtis dissimilarity matrix. ANOSIM was carried out to assess similarity between sampling location types and seasons; this was done using the “anosim” function from the vegan package version 2.4-3 (Oksanen et al., 2015). The rarefied dataset was also used to test similarity between technical replicates with hierarchical clustering using the “hclust” function in R version 3.3.0 of Bray–Curtis dissimilarities between samples found using the “vegdist” function in vegan.

Bray–Curtis dissimilarities between pig stables and associated farmer’s homes (i.e., the sample pair reflecting where a given farmer worked and lived) were calculated using the “distance” function in phyloseq version 1.16.2. The similarity between each associated stable–home pair was ranked among all non-matching stable–home pairs, with the resulting rank reflecting how similar associated stable–home pairs were compared to random association between any pig stable and any non-associated farmer’s home. A rank of one would be a strong indication of significant bacterial transfer between the farmer’s home and the pig stable where he or she works; a random ranking would signify no association.

A significance level of *P* < 0.05 was used for all statistical tests.

### Accession Numbers

MiSeq-derived sequences used in this study have been deposited in the NCBI sequence read archive (SRA) under study number SRP124427.

## Results

### Sample Collection Time

The targeted EDC sample collection time was 14 days, with 45% of samples collected for 14 days, 32% of samples collected for more than 14 days, 18% of samples collected for less than 14 days, and 5% of samples with a collection time unrecorded by the volunteer household. Recorded collection times ranged from 7 to 73 days. There were few significant differences between the distributions of collection times for each sample type (Supplementary Figure 1). Sample collection times did not have a significant positive correlation with bacterial abundances measured by qPCR (Supplementary Figure 2) nor showed any effect on the observed community composition that could affect the conclusions of this study (Supplementary Figure 3).

### Bacterial Abundance

**FIGURE 1:**
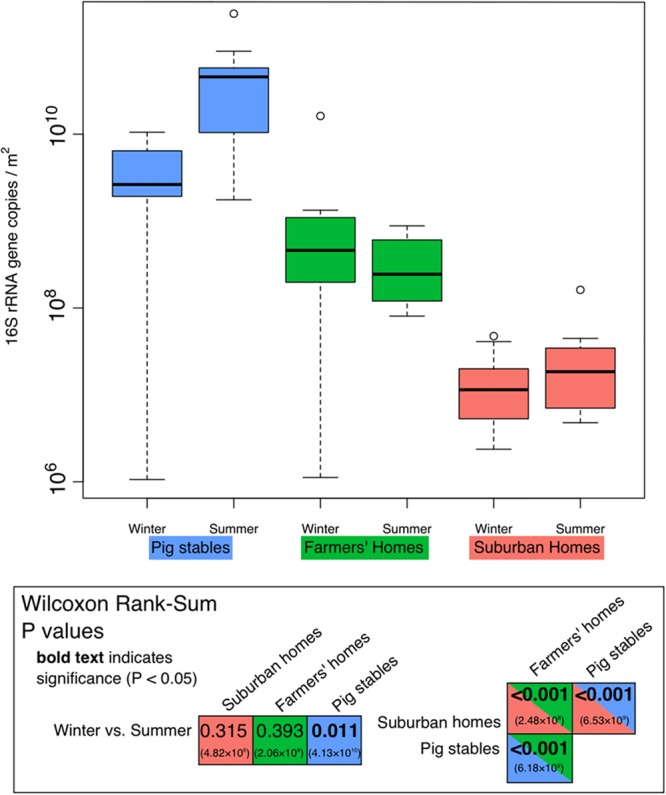
Quantitative PCR measurements of the 16S rRNA genes in each environment type/season using bacteria-specific primers. Units are 16S rRNA gene copies per m^2^ of EDC (following 14 days of exposure). Each season/type box represents all 10 measurements made. Significance of the differences shown in this figure is demonstrated in the inset box containing results of the Wilcoxon rank sum test for various comparisons between seasons or environment types. *P* values in bold indicate values below 0.05 (significant). Values in parentheses are absolute values of Hodges–Lehmann estimators, showing the magnitude of the difference between the two sample types.

Bacterial 16S rRNA genes were most abundant in dust collected from pig stables, followed by farmers’ homes and suburban homes (**Figure 1**). All differences in abundance between location types were significant (*P* < 0.001). A significant seasonal difference was only observed for pig stables, where higher abundances were observed in summer samples (*P* = 0.011). Although 16S rRNA gene copies per genome can vary from 1 to 15 (Vétrovský and Baldrian, 2013), our observed differences, spanning four orders of magnitude, were greater than what could be explained by copy number variation alone.

### 16S rRNA Gene Sequencing Reproducibility

Of 15 technical replicate pairs included in the final analysis, 13 replicates clustered most closely with their replicate partner, while two were clustered elsewhere (Supplementary Figure 4). This indicates that the methodology used to extract DNA and analyze bacterial community composition was largely reproducible across different samples. This reproducibility confirms the validity of between-sample comparisons of diversity and composition in this dataset. Technical replicates were not included in subsequent statistical tests, with one of the two replicates chosen arbitrarily to represent a given sample.

### Alpha and Beta Diversity in Bacterial Airborne Communities

**FIGURE 2:**
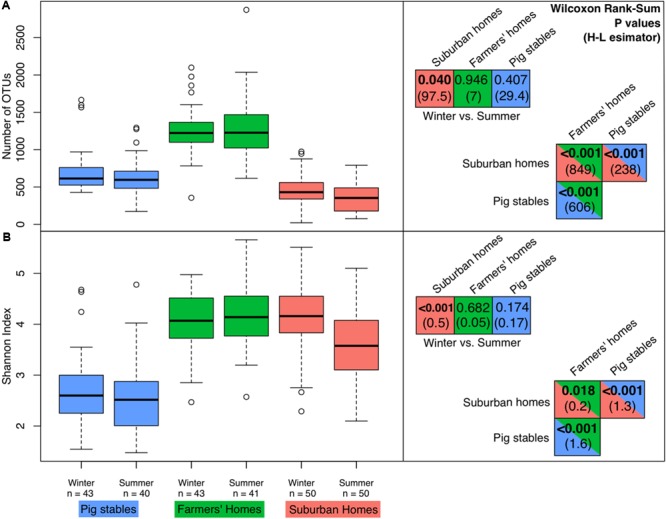
Diversity measures in the different environment types/seasons. “*n*” indicates the numbers of samples for each environment type/season (technical replicates not included in this analysis). The right-hand panels show *P* values from Wilcoxon rank sum tests comparing richness/Shannon index in different environment types and seasons. Values in parentheses are absolute values of Hodges–Lehmann estimators, showing the magnitude of the difference between the two sample types. Bold *P* values are less than 0.05 (significant differences). **(A)** Boxplots of richness in terms of number of OTUs. **(B)** Boxplots of the Shannon index (taking into account both richness and evenness, with higher numbers indicating higher diversity).

**FIGURE 3:**
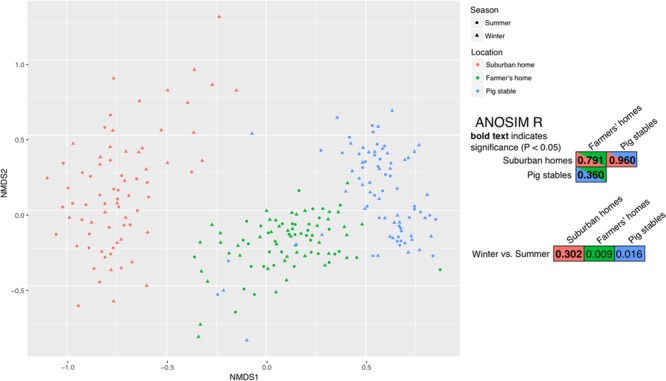
Non-metric multidimensional scaling (NMDS) for all samples with 8000 or greater reads. The right-hand panel shows the ANOSIM R metric, a number indicating the degree of difference between the sample types, for comparisons of seasons and environment types. *R* = 1.0 means very different bacterial community composition and *R* = 0.0 means very similar bacterial community composition. Bold text indicates a significant difference (*P* < 0.05).

In terms of richness (numbers of OTUs), farmer’s homes had the highest bacterial richness in their collected dust, followed by pig stables, followed by suburban homes (**Figure 2A**). All of these differences in richness were significant (*P* < 0.001). A significant seasonal difference in richness was only observed for suburban homes, where greater richness was observed in winter samples (*P* = 0.040). The Shannon index, which takes both richness and evenness into account, also showed the dust collected in farmers’ homes as the most diverse environment type (**Figure 2B**). However, in contrast to richness alone, the Shannon index showed suburban homes as the second most diverse, followed by pig stables as the least diverse. All these differences were significant (max. *P* = 0.018). Seasonal differences in Shannon index matched differences in richness, with only suburban homes showing a significantly greater diversity in winter (*P* < 0.001). The OTU composition of farmers’ homes, suburban homes, and pig stables was significantly different from each other based on the ANOSIM test (**Figure 3**). The difference was greatest between suburban homes and pig stables (ANOSIM *R* = 0.960), with farmers’ homes constituting an intermediate sample type (ANOSIM *R* = 0.360 compared to pig stables and 0.791 compared to suburban homes). A significant seasonal difference was observed for suburban homes (ANOSIM *R* = 0.302), but not farmers’ homes or pig stables. All of these differences were evident in the spatial organization of samples plotted in the non-metric dimensional scaling (NMDS) ordination (**Figure 3**).

### Bacterial Community Composition and Differential Abundance

**FIGURE 4:**
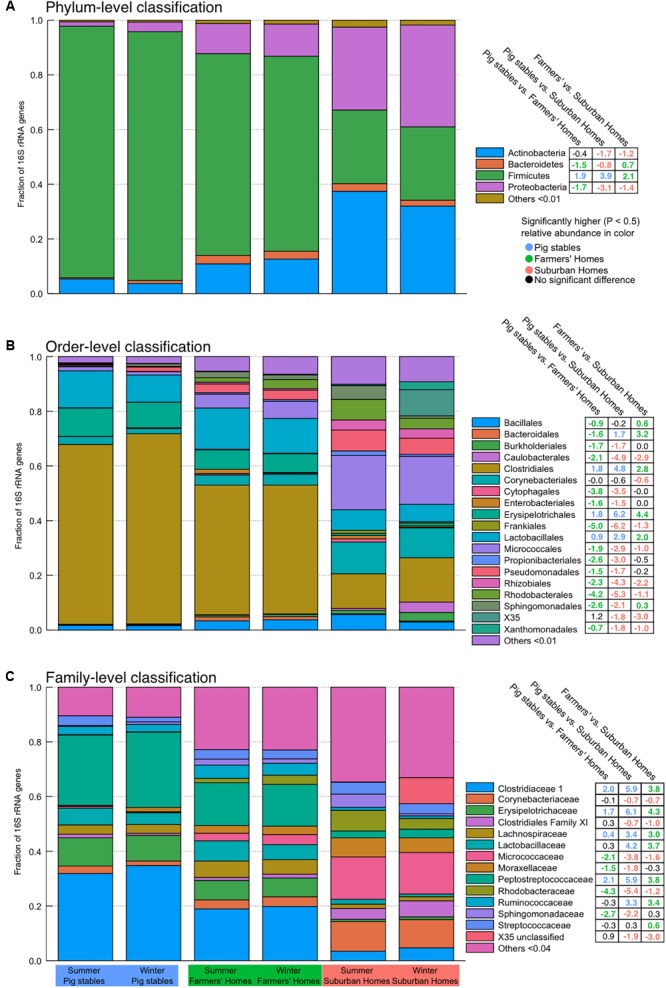
Stacked bar plots indicating community-level composition at phylum **(A)**, order **(B)**, and family **(C)** levels. Abundances are weighted such that each sample contributes equally to the relative fraction illustrated. Values in the tables on the right-hand side are derived from the ALDEx2 analysis of differential abundance and show the median difference in centered log-ratio (clr) values for that particular taxon, illustrating the magnitude of the difference in abundance. Significant differences have colored text (colored according to the environment type) and non-significant differences have black text. Positive numbers mean the environment type listed before “vs.” contains a greater fraction of that particular taxon and negative numbers mean the environment type after “vs.” contains a greater fraction of that particular taxon.

All sample types consisted almost entirely of four bacterial phyla: Firmicutes, Proteobacteria, Actinobacteria, and Bacteroidetes (**Figure 4A**). Firmicutes were in especially high relative abundance in samples collected in pig stables, with a progressively smaller fraction found in farmers’ homes and suburban homes. Instead of Firmicutes, dust collected in homes contained significantly larger fractions of Proteobacteria, Actinobacteria, and Bacteroidetes. These trends in phylum abundance were found to be significant by ALDEx2 analysis with the exception of Actinobacteria abundance, which was not significantly different between pig stables and farmers’ homes (Supplementary Tables 1–3). Order-level classification showed the predominance of bacteria from the order Clostridiales, especially in pig stables and farmers’ homes (**Figure 4B**). There were significantly more Clostridiales in samples from pig stables and farmers’ homes than in suburban homes (Supplementary Tables 7–9). Other orders more abundant in pig stables and farmers’ homes were Lactobacillales and Erysipelotrichales. For family-level classification, farmers’ homes and pig stables were characterized by significantly greater abundances of Clostridiaceae 1, Peptostreptococcaceae, Lachnospiraceae, Erysipelotrichaceae, Lactobacillaceae, and Ruminococcaceae (Supplementary Tables 4–6). These families had progressively greater fractional abundance in farmer’s homes and pig stables compared to suburban homes.

### Similarity of Bacterial Community Composition Between a Farmer’s Home and Pig Stable

We carried out a pairwise analysis of similarity between pig stables and farmer’s homes, to see whether associated pig stables and farmer’s homes (i.e., the pair of locations where the farmer worked and lived) were more similar to each other (Bray–Curtis similarities) than to the overall degree of similarity between pig stables and farmers’ homes (Supplementary Figure 5). We could show that only 3 out of 79 associated stable–home pairs were more similar than non-associated stable–home pairs. It was more likely that the indoor air bacterial community in a given farmer’s home was more similar to the indoor air bacterial community in another farmer’s pig stable than in his or her own pig stable.

## Discussion

Our results show that the three environments investigated: pig stables, farmers’ homes, and suburban homes, can be distinguished from one another based on the abundance, diversity, and community composition of their airborne bacteria. All of the observed differences in bacterial community composition and abundance of specific types of bacteria support the hypothesis that airborne bacteria in farmers’ homes, and to some extent pig stables, may contribute to protection against asthma and allergy. We suggest that in future studies on the association between the composition of microbial communities and the prevalence of asthma and allergies, microbiome studies should be combined with epidemiological studies on comparable cohorts.

### Elevated Airborne Bacterial Abundance in Farmers’ Homes and Pig Stables

We found significantly higher bacterial 16S rRNA gene copies in pig stables and farmers’ homes (**Figure 1**) than in suburban homes. Several studies have shown a link between increased bacterial abundance and protection against atopy. These studies have mostly been based on the measure of endotoxin (Portengen et al., 2005) [also known as lipopolysaccharide (lipid A), a component of the outer membrane in Gram-negative bacteria] or muramic acid (a component of bacterial cell walls, especially abundant in Gram-positive bacteria; van Strien et al., 2004; Pakarinen et al., 2008). Gram-positive (e.g., Firmicutes and Actinobacteria) and Gram-negative (Proteobacteria and Bacteroidetes) bacteria were both identified in our samples by 16S rRNA gene sequencing (**Figure 4A**). The aerosolization of microbes associated with pig skin, epithelial tissues, and excretions results in a large indoor source of bacterial cells and can likely explain greater bacterial abundances in pig stable air than in the two other environments. This is consistent with previous studies that show elevated bacterial abundances in pig stable air relative to control indoor environments (Hong et al., 2012), with pig feces as the primary source of elevated airborne bacteria density (Kristiansen et al., 2012; Kumari and Choi, 2015). Greater bacterial abundance in the air of farmers’ homes compared to suburban homes can likely be explained by a greater variety of outdoor microbial sources in rural areas compared to suburban areas, such as soil, plants, water, and animals. For example, air downwind of vegetated areas has been shown to have significantly higher bacterial concentrations relative to non-vegetated areas (Lymperopoulou et al., 2016). Outdoor air and human presence have been shown to be the two major sources for indoor airborne bacteria (Adams et al., 2015a), so increased bacterial numbers in outdoor air could reflect increased numbers in indoor air.

### Elevated Airborne Bacterial Diversity in Farmers’ Homes

The richness we observed in homes ranged from hundreds of OTUs to the 1000–2000 OTU range (**Figure 2**). This is consistent with previous measurements of bacterial richness in homes (Ege et al., 2011; Dannemiller et al., 2015; Kettleson et al., 2015). Increased airborne bacterial diversity in homes has been linked to a lower prevalence of asthma and allergy (Pakarinen et al., 2008; Ege et al., 2011; Lynch et al., 2014; Haahtela et al., 2015). Our results showed the highest diversity in farmers’ homes compared to suburban homes and pig stables. To our knowledge, this is the first study to demonstrate that farmer’s homes have a higher diversity of airborne bacteria than pig stables, suggesting that the putative protective effect of higher airborne bacterial diversity will be strongest in a farm home rather than in a pig stable. This contrasts with previous research showing a protective effect associated with presence in stables (Illi et al., 2012), but that study found that this effect was only significant in cow stables and not pig stables. Interestingly, the difference between farmers’ homes and suburban homes was much greater when comparing the number of OTUs than comparing the Shannon index (**Figure 2**). Since the Shannon index takes both richness and evenness into account, this shows that the additional richness in farmers’ homes is not evenly distributed in abundance compared to the rest of the community. This suggests that the higher richness in farmers’ homes is a “long tail” of low-abundance OTUs missing from suburban homes. Additional low-abundance diversity would be consistent with that diversity originating from an outside source following dilution in outside air, or as contaminants brought in from the stables. This is consistent with previous research showing higher bacterial diversity in rural and remote areas compared to cities (Bowers et al., 2011a,b). The relatively low diversity of airborne bacteria in pig stables can be explained by the closed and highly controlled nature of the pig stable environment with low genetic diversity among the animals, the application of antibiotics, mechanical ventilation, and the buildings’ single use, meaning that bacteria in the air will to a greater extent come from a single source, the pigs and their feed. This is consistent with the identification of pigs and their excretions as the primary source for airborne bacteria in pig stables as shown in previous studies (Nehme et al., 2008; Kristiansen et al., 2012; Kumari and Choi, 2015).

### Bacterial Community Composition

The community composition of pig stables was largely consistent with prior studies of the pig stable airborne bacterial community, with the phylum Firmicutes as the most abundant relative to other phyla (Kristiansen et al., 2012; Kumari and Choi, 2014, 2015). The higher abundance of Firmicutes relative to farmers’ homes and suburban homes is also consistent with a previous comparison between pig stables and offices (Hong et al., 2012) or pet-free households (Boissy et al., 2014). In all environment types, we found certain taxonomic groups of bacteria that potentially cause a protective effect against asthma and allergy when present in the indoor environment. These include Gram-positive bacteria (van Strien et al., 2004; Pakarinen et al., 2008) such as phyla Firmicutes and Actinobacteria, and the families Prevotellaceae, Lachnospiraceae, and Ruminococcaceae (Lynch et al., 2014), and Peptostreptococcaceae (Pekkanen et al., 2018). With the exception of Actinobacteria, there was a significantly higher relative abundance of all these families and phyla in our pig stable and farmers’ homes samples than in suburban homes (**Figure 4** and Supplementary Tables 1–6). There is experimental evidence from mouse models that exposure to a species within the family Ruminococcaceae, *Clostridium leptum* (also known as *Ruminiclostridium leptum*; Yutin and Galperin, 2013), helps to prevent the development of allergy and asthma (Li et al., 2012; Huang et al., 2015). This same observation was made in a study with mice exposed to *Lactobacillus reuteri* (Forsythe et al., 2007; Karimi et al., 2009). In the present study, the genus that *C. leptum* belongs to (designated “Ruminiclostridium_5” in the SILVA database version 128; Quast et al., 2013) and *Lactobacillus* were both found to have a significantly higher relative abundance in farmers’ homes compared to suburban homes (Ruminiclostridium_5 log_2_ fold difference = 3.8, BH adjusted *P* < 0.001, *Lactobacillus* log_2_ fold difference = 3.6, BH adjusted *P* < 0.001; Supplementary Table 12) and in pig stables compared to suburban homes (Ruminiclostridium_5 log_2_ fold difference = 2.6, BH adjusted *P* < 0.001, *Lactobacillus* difference = 3.7, BH adjusted *P* < 0.001, Supplementary Table 11). A significant difference was found in relative abundance between pig stables and farmers home for Ruminiclostridium_5, but not for *Lactobacillus* (Ruminiclostridium_5 log_2_ fold difference = -1.2 BH adjusted *P* = 0.02, *Lactobacillus* difference = 0.11, BH adjusted *P* = 0.83, Supplementary Table 10).

The majority of studies cited above found correlations between the abundance of certain bacterial taxa and allergic disease without directly identifying the direct causal factor. There is of course the possibility that an unmeasured factor is influencing both airborne bacterial communities and the development of allergic disease, and that the bacteria themselves are not the causal factor. However, the experimental studies carried out with *C. leptum* and *L. reuteri* demonstrate a direct causal link between exposure to specific bacteria and protection. This means that a causative relationship between our observed bacterial communities in farmers’ homes and pig stables and the conferral of atopic protection is an intriguing possibility that merits further investigation.

### Negative Health Consequences From Farm-Based Airborne Bacteria

While living on farms apparently provides atopic protection, farms also cause negative health outcomes that may also be related to the airborne bacterial community. While higher airborne endotoxin concentrations (i.e., higher abundances of Gram-negative bacteria) have been found to protect against atopic asthma, they have also been shown to increase the risk of non-atopic asthma (Eduard et al., 2004; Sigsgaard et al., 2005). Other negative health consequences apart from asthma or atopy include COPD and chronic bronchitis (Monsó et al., 2004; Eduard et al., 2009). Genera containing harmful pathogens associated with pig stables, such as species within the genus *Staphylococcus* (Masclaux et al., 2013), were found in all environments. Although their fractional abundance was not significantly greater in pig stables and farmers’ homes than suburban homes (Supplementary Tables 10–12), the overall higher abundance of bacteria in pig stables and farmer’s homes suggests a higher absolute number of *Staphylococcus* sp. present in the air. Higher abundance of airborne *Staphylococcus* in pig stables leads to higher rates of nasal carriage among farm workers (Bos et al., 2016). We found airborne bacterial concentrations 10–100× higher in pig stables compared to farmers’ homes, suggesting that farmers in pig stables are exposed to a higher concentrations of potentially hazardous bacteria than in their homes, with *Staphylococcus* as an example.

### Seasonal Effects on Bacterial Community Composition and Abundance

Seasonal differences in airborne bacterial abundance, diversity, and composition were limited. Abundance differed seasonally in pig stables only, with higher bacterial concentrations present in summer compared to winter, likely a result of increased temperature and humidity in the summer months. This is in contrast to previous studies showing greater bacterial abundance in Canadian (Nehme et al., 2008) and South Korean (Kumari and Choi, 2014) pig stables during winter. This difference may reflect different agricultural practices in South Korea, Canada, and Denmark and needs to be investigated further. Diversity and composition showed seasonal variation in suburban homes, but not in pig stables or farmers’ homes. Seasonal stability in pig stables can be explained by the tightly controlled and closed environment, with mechanical ventilation, uniform activities, and stable feed composition all year-round. The constant diversity and composition between seasons in this study was in contrast to what was found in a study of South Korean pig stables (Kumari and Choi, 2014). We suggest two possible explanations for this difference: (1) different agricultural practices in different countries or (2) greater statistical certainty derived from the larger number of pig stables sampled in this study (seven pig stables in the Korean study vs. 43 in our Danish study). While the findings in the Korean study contradict our findings, a constant diversity between summer and winter periods is consistent with an earlier seasonal comparison of bacterial diversity in Canadian pig stables (Nehme et al., 2008). We interpret the seasonal consistency in farmers’ homes as a sign of more consistent usage patterns around the year, possibly related to cleaning and ventilation activities. Members of bacterial families that were more abundant in suburban homes in winter included the so-called “X35” family, Clostridiales XI, and Clostridiaceae 1, while during summer, members of the Sphingomonadaceae and Rhodobacteraceae were more abundant in suburban homes (**Figure 4**). A previous study of human-home microbiota transfer showed that X35, Clostridiales XI, and Clostridiaceae 1 (Clostridia and Gammaproteobacteria) that are classified within classes more likely to originate from humans, while Sphingomonadaceae and Rhodobacteraceae (Alphaproteobacteria) were more likely to originate from the surrounding environment (Lax et al., 2014). This means that the winter airborne bacterial community in suburban homes is more likely to be derived from the occupants, while the summer airborne bacterial community has more outdoor inputs. This is consistent with open windows, greater air circulation, and more traffic in and out of the home during summer.

### Paths of Exposure

The majority of studies suggest that the protective effect of airborne bacteria exposure against asthma and allergy is highest if the exposure takes place at a very young age (von Mutius and Vercelli, 2010), with the effect being stronger at <1 years compared to 1–5 years of age (Riedler et al., 2001), or even during pregnancy (Illi et al., 2012). We have shown that there is no significant direct transfer of bacteria from the air of pig stables to the farmers’ homes, which suggests that the protective exposure must be found within the farm homes. This may be a result of Danish regulations that prescribe strict rules for employees exiting pig stables including hand-washing, changing clothes, and disinfection of equipment to prevent transmission of zoonotic pathogens (Bek nr 534 af 27/05/2014, 2014). This means that the putatively beneficial or detrimental bacteria in the air of farm homes do not come directly from the pig stable as a contamination associated with each particular farm home. This also suggests that the comparison between farmers’ homes and suburban homes is more relevant for understanding the preventative effects on asthma and allergy than comparison to the pig stables. On the other hand, there is also evidence that the farm environment can confer protection in adults (Portengen et al., 2002; Elholm et al., 2013, 2016), so a protective effect for older children or adults regularly exposed to air in pig stables cannot be ruled out. If we accept that living in a farm home will have a greater protective effect than living in a suburban home, then where does this protective effect come from? As described earlier, the additional diversity present in farm homes compared to the suburban homes is likely due to outdoor sources, suggesting that putatively beneficial or detrimental bacteria in indoor farm home air are transported from the source environments surrounding the farmers’ homes rather than from the farmer’s own pig stables. The fact that farm homes are located in open areas with lower urban density could be part of the explanation, as an inverse correlation between airborne bacterial diversity and the percentage of built area (i.e., degree of urbanization) has been identified (Parajuli et al., 2018). Additionally, farms are unique open spaces that include microbial sources not present in other types of open space. For example, pig manure is commonly stored in uncovered tanks or spread on agricultural soil in Denmark, and a link between pig gut microbiota and the manure-impacted soil microbial community has been identified (Graham et al., 2016). This suggests an indirect transfer of microbes from the pigs to the farm home air, which might explain putatively beneficial bacteria common to the air of both farmers’ homes and pig stables. Future research into the transfer of bacterial communities from farm-based bacterial sources other than pig stable air, such as animals and their excrement, plant surfaces, soil, storage facilities, and surface water, is necessary to determine the source of putatively beneficial bacteria in the indoor air of farmer’s homes.

## Conclusion

Previous research has shown that being born and raised on a farm confers a degree of protection against the development of asthma and allergies, with the airborne bacterial community exposure as a proposed explanatory factor. Our study of Danish pig stables, associated farm homes, and suburban homes supports this hypothesis, with higher airborne bacterial abundance in pig stables and farmers’ homes, and higher diversity in farmers’ homes compared to suburban homes. Moreover, specific bacterial taxa with a putative protective effect against the development of asthma and allergy were found at a significantly greater abundance in pig stables and farmers’ homes compared to the controls. There is, however, no obvious connection between the airborne bacterial community in a farmer’s home and his or her pig stable, suggesting that any beneficial or detrimental effect of the airborne bacterial community in a farm home is not directly derived from the adjacent pig stable. This study identifies putatively protective airborne bacterial community characteristics in farmers’ homes and pig stables to a greater extent than in suburban homes. It further supports the hypothesis that farm exposure protects against the development of asthma and allergy through the presence of their airborne bacterial communities. In future studies, investigations into the microbiome of production facilities and homes should be linked to epidemiological studies on the prevalence of asthma and allergy among subjects that occupy these environments. In addition, the causes of the microbiome-derived protective effects need to be elucidated to find ways of averting the asthma and allergy pandemic.

## Author Contributions

IB, GE, VS, and TS designed and carried out sample collection from pig stables and farmers’ homes under the SUS12 study. GH, AL, and TS designed and carried out sample collection for suburban homes. DV, GH, KF, TS, TŠ-T, and IM planned qPCR and 16S rRNA gene sequencing. DV and GH extracted cells from EDC filters. DV extracted DNA from cells and carried out PCR and sequencing. DV and IM analyzed the data from sequencing and qPCR and wrote the manuscript. All authors revised the manuscript.

## Conflict of Interest Statement

The authors declare that the research was conducted in the absence of any commercial or financial relationships that could be construed as a potential conflict of interest.
