# Supplementary material for: Pig Farmers’ Homes Harbor More Diverse Airborne Bacterial Communities Than Pig Stables or Suburban Homes
Source: Front Microbiol. 2018 May 1;9:870. doi: 10.3389/fmicb.2018.00870 (PMC5938556; doi:10.3389/fmicb.2018.00870)
Supplement: Supplementary file 2 [file Image_1.PDF]

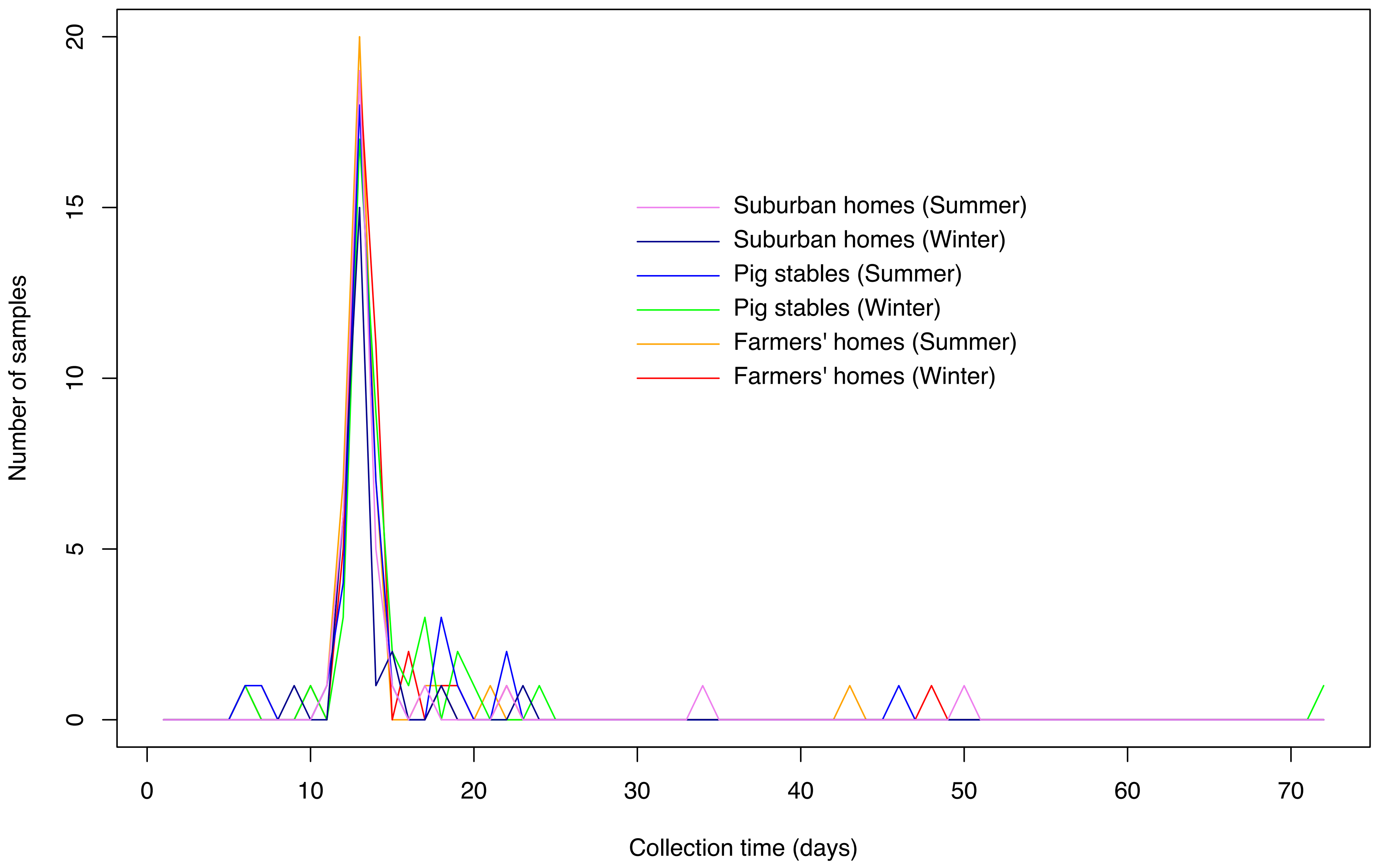

Supplemental Figure SF1 - Distribution of collection times for all sample types. Almost all distributions were not significantly different from one another based on the Wilcoxon Rank Sum Test. The exceptions were pig stables in winter compared to farmer's homes in summer (P = 0.03) and suburban homes in winter (P = 0.02).

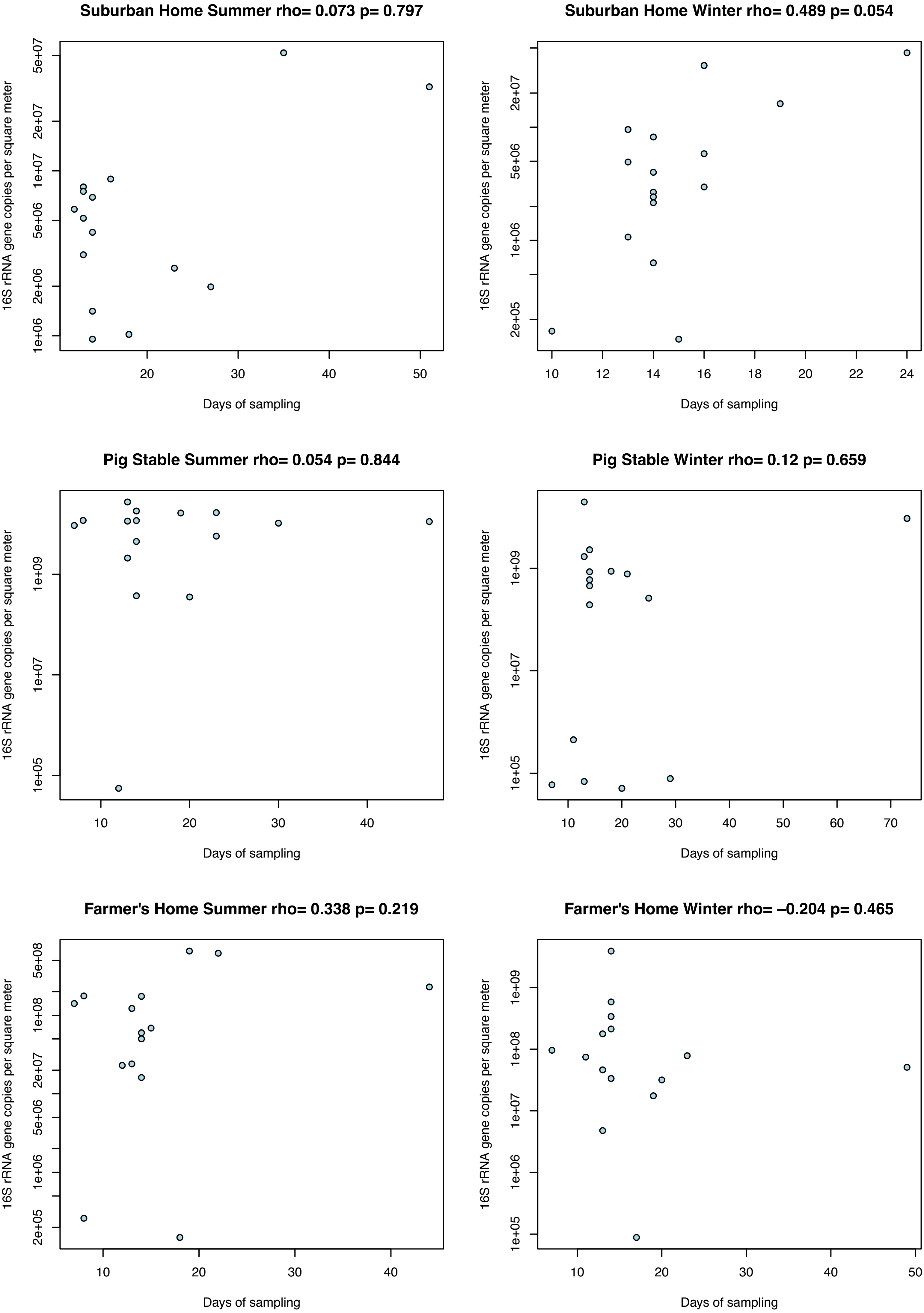

Supplemental Figure SF2 - Spearman correlation analysis for qPCR data and collection times. Spearman's rho and p values provided for each location type / season.

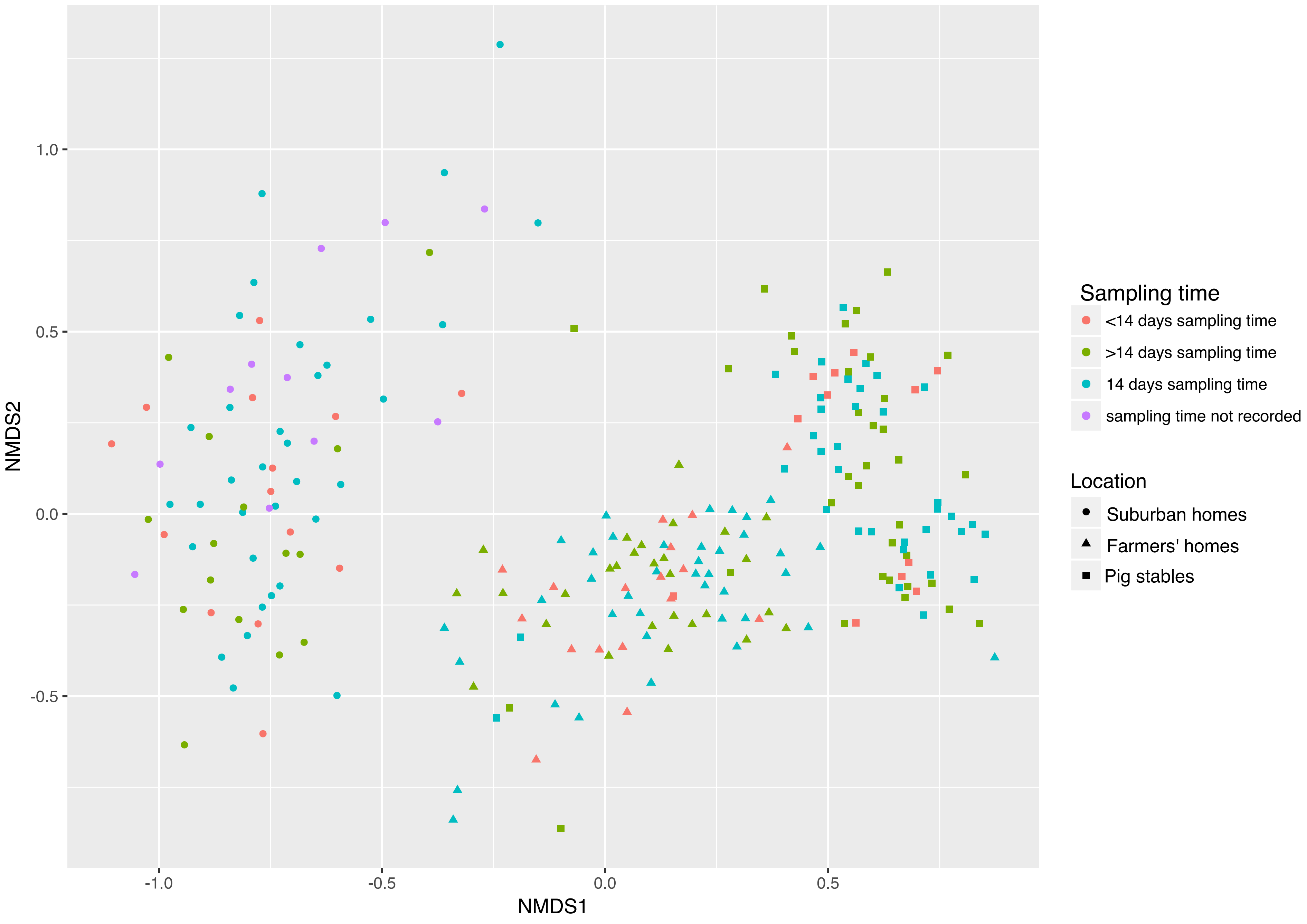

Supplemental Figure SF3 - NMDS analysis (corresponds to figure 3) colored by sampling time.

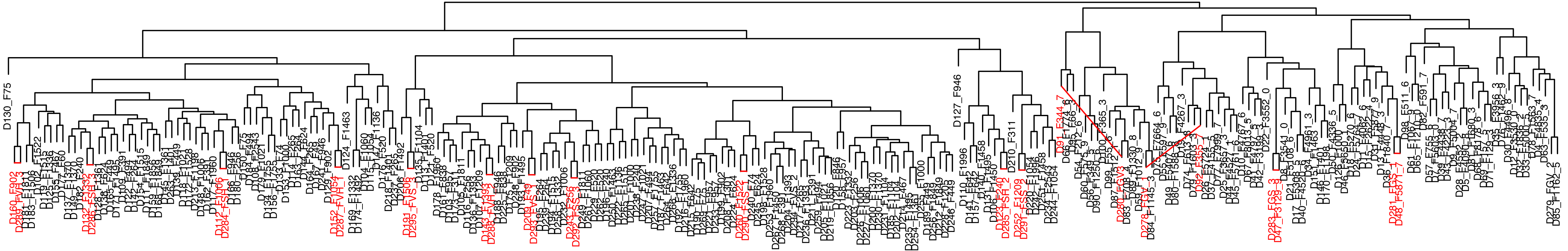

Supplemental Figure SF4: Hierarchical clustering of all samples in this study including technical replicates based on a Bray-Curtis dissimilarity matrix. Samples and technical replicates are indicated with red text, and red lines between leaves connect each replicated sample and its replicate.

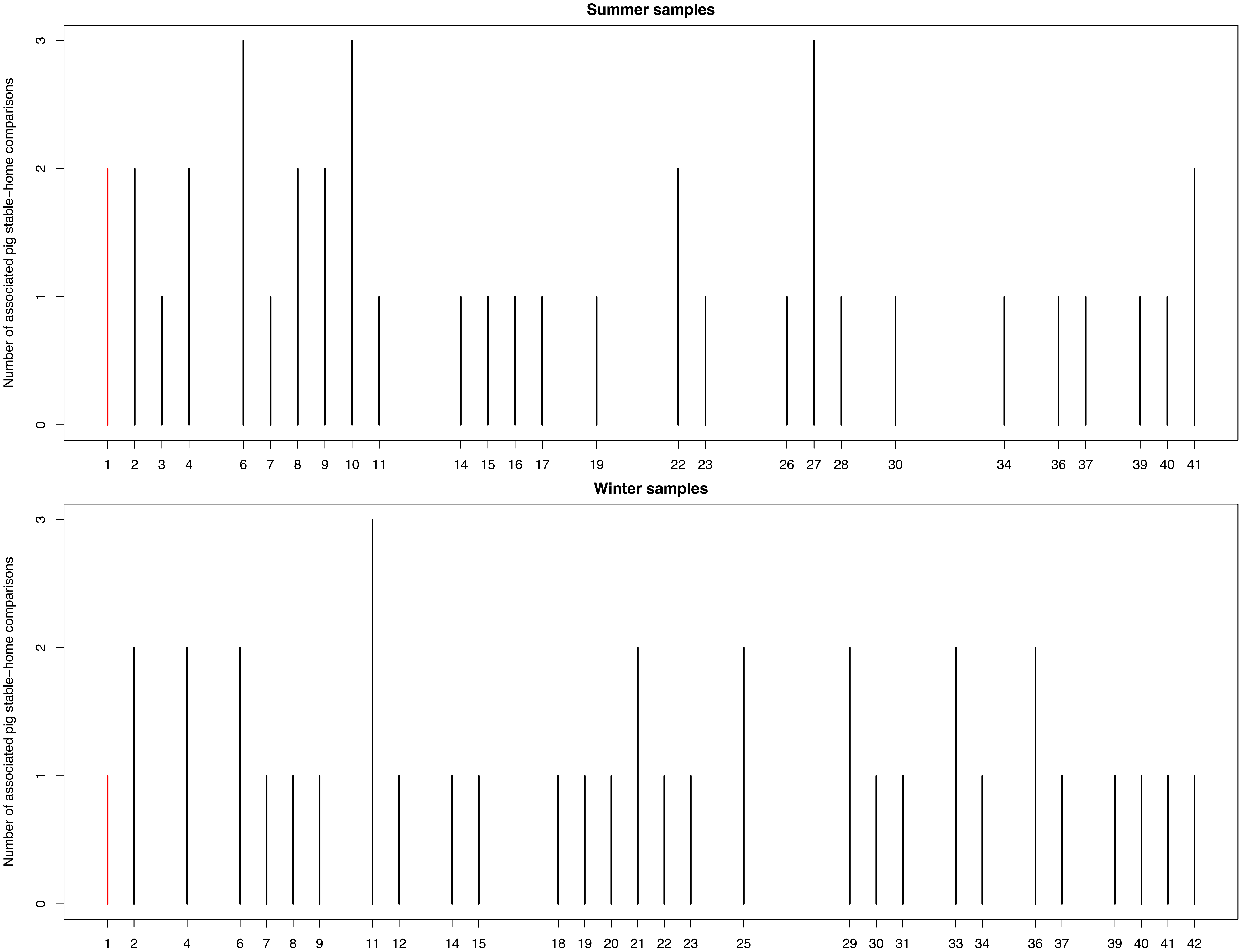

Supplemental Figure SF5 : Plots showing the similarity rank of associated pig stable-home pairs in a ranked list of all possible pig stable-home pairs. A similarity rank of 1 (colored red) indicates that the airborne bacterial community in a given farmer's home is more similar to the pig stables where that farmer works than any other pig stable.
